# WHO informal consultation on revision of guidelines on evaluation of similar biotherapeutic products, virtual meeting, 30 June – 2 July 2021

**DOI:** 10.1016/j.biologicals.2022.03.001

**Published:** 2022-04

**Authors:** Meenu Wadhwa, Hye-Na Kang, Robin Thorpe, Ivana Knezevic, following participants of the WHO informal consultation on revision of guidelines on evaluation of similar biotherapeutic products, P. Aprea, M.-C. Bielsky, N. Ekman, H.-K. Heim, J. Joung, P. Kurki, E. Lacana, C. Njue, E. Nkansah, M. Savkina, R. Thorpe, T. Yamaguchi, M. Wadhwa, J. Wang, M. Weise, E. Wolff-Holz, Other participants, M. Allam, H. Bahaa, M. Sayed, A. Al-Oballi, A. Alshahrani, D. Baek, J. Kim, H.M. Chua, J. Gangakhedkar, Mr P. Jagtap, T. Lyaskovsky, S. Okudaira, W. Ondee, P.S. Sotomayor, J.I. Solis Ricra, J. Uviase, Representatives of the Developing Countries Vaccine Manufacturers Network, F. Ahmed, Y. Rajendran, H.G. Tonioli Defendi, Representatives of the Emerging Biopharmaceutical Manufacturers Network, S.Yi O. Cho, A. Qu, Representatives of the IFPMA, V. Acha, M. Gencoglu, K. Ho, Representatives of IGBA, M. Baldrighi, M. Schiestl, K. Watson, Representative of the Latin American Association of Pharmaceutical Industries, E. Spitzer, Representative of the Singapore Association of Pharmaceutical Industries, S. Chong, WHO Secretariat, A. Fukushima, H.-N. Kang, I. Knezevic, G. Pante, Mariangela Simao

**Affiliations:** h Ministry of Food and Drug Safety, Republic of Korea; o Pharmaceuticals and Medical Devices Agency, Japan; s Egyptian Drug Authority, Egypt; t Jordan Food and Drug Administration, Jordan; u Saudi Food & Drug Authority, Saudi Arabia; v Malaysia National Pharmaceutical Regulatory Agency, Malaysia; w Central Drugs Standard Control Organisation, India; x Ministry of Health of Ukraine, Ukraine; y Ministry of Public Health, Thailand; z Ministry of Health, Peru; aa National Agency for Food and Drug Administration and Control, Nigeria; ab Incepta Pharmaceuticals Ltd, Bangladesh; ac Zydus Cadila Healthcare, India; ad R&D Biomanguinhos, Brazil; ae Instituto Butantan, Brazil; af China National Biotec Group Company Ltd, China; ag MSD, Switzerland; ah IFPMA, Switzerland; ai F. Hoffmann-La Roche Ltd, Switzerland; aj Medicines for Europe, Belgium; ak Sandoz Biopharmaceuticals, Austria; al Celltirion, Republic of Korea; am Latin American Association of Pharmaceutical Industries, Argentina; an Singapore Association of Pharmaceutical Industries, Singapore; ao Access to Medicines and Health Products, World Health Organization, Switzerland; a National Institute for Biological Standards and Control, Medicines and Healthcare Products Regulatory Agency, Potters Bar, United Kingdom; b World Health Organization, Department of Essential Medicines and Health Products, Avenue Appia 20, CH-1211, Geneva, Switzerland; c Independent Expert, Welwyn, United Kingdom; d Administración Nacional de Medicamentos, Alimentos y Tecnología Medica, Argentina; e Medicines and Healthcare Products Regulatory Agency, United Kingdom; f Finnish Medicines Agency, Finland; g Federal Institute for Drugs and Medical Devices, Germany; h Ministry of Food and Drug Safety, Republic of Korea; i University of Helsinki, Finland; j United States Food and Drug Administration, USA; k Health Canada, Canada; l Food and Drug Authority, Ghana; m Federal State Budgetary Institution Scientific Centre for Expert Evaluation of Medicinal Products, Russian Federation; n Independent Expert, United Kingdom; o Pharmaceuticals and Medical Devices Agency, Japan; p National Institute for Biological Standards and Control, Medicines and Healthcare Products Regulatory Agency, United Kingdom; q National Institutes for Food and Drug Control, China; r Paul-Ehrlich-Institut, Germany

**Keywords:** Biosimilar, Similar biotherapeutic product, Regulatory guidelines, Revision, WHO

## Abstract

The WHO informal consultation was held to promote the revision of WHO *guidelines on evaluation of similar biotherapeutic products (SBPs)* adopted by the Expert Committee on Biological Standardization (ECBS) in 2009. It was agreed in the past consultations that the evaluation principles in the guidelines are still valid, but a review was recommended to provide more clarity and case-by-case flexibility. The opportunity was therefore taken to review the experience and identify areas where the current guidance could be more permissive without compromising its basic principles, and where additional explanation could be provided regarding the possibility of reducing the amount of data needed for regulatory approval. The meeting participants applauded the leading role taken by the WHO in providing a much-needed streamlined approach for development and evaluation of SBPs which will provide efficient and cost-effective product development and increase patient access to treatments. It was recognized that the principles as currently described in the draft WHO guidelines are based on sound science and experience gained over the last fifteen years of biosimilar approvals. However, since these guidelines when finalised will constitute the global standard for biosimilar evaluation and assist national regulatory authorities in establishing revised guidance and regulatory practice in this complex area, it was felt that further revision and clarity on certain perspectives in specific areas was necessary to dispel uncertainties arising in the current revised version. This report describes the principles in the draft guidelines, including topics discussed and consensus reached.

## Highlights

• WHO guidelines serve as a basis for the development of national regulatory framework for biosimilars.
• Revision of guidelines is to provide more flexibility and clarification on data required for regulatory approval.
• Revised guidelines would contribute to improving consistency on regulatory decision and patient access to treatments.

## 1 Introduction

The approval of the first biosimilar fifteen years ago in Europe brought promise of safe and effective treatments at reduced costs to patients lacking access to affordable life-saving medicines. Today while we build on the expansion in biotechnological products with the success of many biosimilar approvals, this promise is still to be realised in many countries in the world. The WHO responded very rapidly to this news and worked efficiently and tirelessly to produce a global written standard for biosimilars with the adoption of the WHO *guidelines on the evaluation of similar biotherapeutic products* (SBPs or biosimilars) by the WHO Expert Committee on Biological Standardization (ECBS) in 2009 [1]. Since then, WHO has continued to provide tremendous help to member states in implementing the evaluation principles in the guidance into regulatory practices in support of the World Health Assembly (WHA) resolution (WHA 67.21) on promoting access to biotherapeutic products with assured quality, safety and efficacy [2]. In parallel, the WHO guidelines have been reviewed regularly and complemented by generating additional guidance where needed, for example*, Guidelines on the evaluation of monoclonal antibodies as similar biotherapeutic products* in 2016 [3] and the document on *Questions and Answers: similar biotherapeutic products* in 2018 [4].

**Dr Mariângela Simao** (WHO Assistant Director-General, Switzerland) opened the meeting and warmly welcomed all the participants to the informal consultation on revision of guidelines on the evaluation of SBPs. She recognized the significant behind the scenes invaluable contribution of the WHO ECBS towards maintaining the quality of biological medicines and expressed her heartfelt thanks to them and other experts, facilitators, participants from regulatory agencies, industry, the WHO secretariat for their help and commitment to science led revision of the guidelines. The intention is that the revised guidelines provide a sufficiently high standard while improving access to safe and efficacious SBPs for treatment as per WHA resolution 67.21 [2]. While acknowledging the extensive contribution of the current WHO guidelines on the evaluation of SBPs [1] in the development of national regulatory frameworks for the licensure of such products, she mentioned that the ECBS at its 72^nd^ meeting (October 2020) recommended that the current scientific evidence and experience gained in the regulatory evaluation of SBPs be reviewed to inform the prospective updating and revision of the 2009 WHO guidelines [5]. This feedback provided an opportunity to review and identify areas where the current guidance could allow flexibility without compromising its basic principles, and where additional explanation could be given on the possibility of reducing the amount of data needed for regulatory approval. In response to the recommendation, work to revise the guidelines was initiated with the first draft of revision posted on WHO Biological website for the first round of public consultation in May 2021. The aim of this informal consultation is to review the main issues raised from the public consultation and to reach consensus to prepare the next draft. The expectation is that the revised guidelines would harmonize requirements worldwide, provide easy and rapid product approval while assuring their quality, safety and efficacy and result in expanding the availability of various product classes as well as more affordable treatment options particularly in low- and middle-income countries.

**Dr Hye Na Kang** (WHO secretariat, Switzerland) welcomed all participants and updated them on two recent significant WHO activities, namely, a) the WHO survey to gain an understanding of the influence of WHO's guidelines over the last decade on medicines regulation particularly in the biotechnology sector and biosimilars globally (to be covered by Dr Thorpe) [6,7] and b) the review of scientific evidence and experience in the regulatory evaluation of biosimilars as well as new developments in response to a request from ECBS in 2020 to identify areas where flexibility could be incorporated in the current guidance without compromising its basic principles, and where additional explanation could facilitate in potentially reducing the amount of data needed for regulatory approval (to be presented by Dr Kurki). She provided information on drafting group members, the timeline for guideline revision and importantly specified that the objectives of the consultation were to exchange experiences and review regulatory advances related to current regulatory approach, discuss main issues arising from the public consultation (first round) including those that are critical and require action in the draft to be prepared for public consultation (second round). Following agreement and consensus opinion, the draft will be modified and finally prepared as a document for ECBS consideration/adoption in April 2022. This will be followed by revision of the *Guidelines on evaluation of monoclonal antibodies as similar biotherapeutic products (SBPs)*, adopted by 2016 ECBS (Annex 2, WHO Technical Report Series, No. 1004) [3] and the document on WHO *Questions and Answers: similar biotherapeutic products* (adopted by 2018 ECBS; Published at WHO web) [4] which complements the general guidance.

**Dr Ivana Knezevic** (Secretary of WHO ECBS, Switzerland) gave an update on WHO standards for vaccines, biotherapeutics, cell and gene therapy products and emphasized the importance of both written and measurement standards in development, licensing and lot release of vaccines and other biological products. To date, WHO has produced 103 documents applicable to biologicals, a majority are vaccine specific (71) with only a few (9) dedicated to biotherapeutics. Over the last few years, with the support of WHO collaborating centres, considerable progress has been made on written and measurement standards for vaccines and biologicals and more recently towards COVID-19 vaccines and monoclonal antibodies for use in treating infectious diseases. Dr Knezevic acknowledged the pivotal role of WHO Collaborating Center, the National Institute of Biological Standards and Control (NIBSC, UK) which provides >90% of biological standards. She highlighted the importance of updating WHO guidelines for biotherapeutic products including SBPs and presented the plan for its revision in 2022 and 2023 which also includes an update of the Guidelines for monoclonal antibodies developed as biosimilars. Efforts undertaken to embed a regulatory framework in WHO member states via implementation workshops at the regional and/or global level with the associated case-studies/publications to enhance knowledge and expertise will continue in coming years.

## 2 Progress and experience over the past ten years

**Dr Robin Thorpe** (WHO consultant, UK) provided feedback on the results of a WHO survey undertaken in 2019 and 2020 by 20 countries from 6 WHO regions who participated in the survey and also the USA. The huge contribution by WHO in establishing guidelines and enabling their implementation at the national level had increased regulatory convergence in some countries as evidenced by adoption of biosimilarity principles and approval of biosimilars and a trend towards increased use of the term ‘biosimilar’ for products (that are genuinely biosimilars). At present, monoclonal antibodies are the dominant product class and in future locally produced biosimilars may become predominant in some countries. Some challenges have been reported at the national level e.g. the requirement for bridging studies to justify use of foreign sourced reference originator products despite lack/insufficient availability of locally licensed/sourced reference biotherapeutic products (RBPs). Lack of sufficient resources for regulatory review and confusion due to co-existence on the market of non-originator and non-biosimilars along with well-regulated products are also common problems. The latter issue is of concern as it is a barrier to biosimilar uptake and erodes confidence in biosimilar use. Other important issues relate to the practice of interchangeability and the naming and labelling of biosimilars. Survey details and outcomes have been published [[6], [7], [8]].

**Dr Pekka Kurki** (WHO consultant, Finland) reported on the outcome of his review of scientific evidence and experience to identify scenarios for reducing nonclinical and clinical data in the guidelines following a request from ECBS (October 2020). His findings and recommendations are as follows: 1) For quality, emphasis on the state-of-the-art demonstration of analytical and functional similarity, particularly the method of defining the acceptance ranges, risk assessment of quality attributes and the role of statistical analysis in defining the acceptance range. 2) For non-clinical evaluation, practices vary in EU and USA - the relevance of standard *in vivo* toxicological for SBPs is questioned. In the EU, stepwise approach with *in vitro* studies initially supported by tox studies if needed based on a sound justification (e.g. novel formulation) is the norm. This can be considered in the revised guidance as *in vitro* testing of the biological function(s) and use of new assays, if validated are increasingly gaining importance especially for antibody-based products. 3) From the clinical perspective, the need for bridging studies and the possibility of using publicly available data when a foreign reference product is used for SBP development instead of the local domestic product (where one exists) should be considered. 4) Current guidance alludes to the waiving of phase 3 efficacy and safety ‘confirmatory’ studies (e.g. small peptides) and the use of surrogate markers as clinical end-points (e.g. filgrastims, enoxaparin, insulin). Further flexibility can be provided by reducing the need for confirmatory efficacy and safety studies as mounting evidence based on the reduced residual uncertainty and current knowledge (long term safety data of biosimilars indicates no concerns - no difference in quality, safety and efficacy of both reference and biosimilar products) and the overall conclusions from three recent publications which assessed the contribution of efficacy and safety studies to benefit/risk evaluation of biosimilars shows a limited role of phase 3 efficacy and safety studies in biosimilarity assessment. However, in cases where these studies are warranted, clear criteria are mandatory and will need to be provided in the guidelines.

## 3 Regulatory perspective on the first draft revision of guidelines

**Dr Mai Allam** (Egyptian Drug Authority, Egypt) explained the legal and regulatory situation in Egypt which since 2016 has been approving an increasing number of biosimilar products annually (1 in 2016; 5 in 2020/21) with product costs averaging 35% lower than the reference product. The guideline on biosimilars elaborated in 2014 was revised in 2020. The Egyptian guideline is based on guidance from WHO and leading regulatory agencies (e.g. EMA, US FDA). The main strengths of the revised WHO guideline were the sections on use of non-local reference biological products, use of WHO international standards and reference reagents which gives confidence in the assays, specifying the number of batches of RBP to be used for the comparability exercise, statistical intervals for the establishment of similarity ranges and the possibility of reduction of clinical data requirements (based on existing principles) which will require emphasis on quality and *in vitro* functional and human pharmacokinetic data. She highlighted issues that arose during evaluation of the biosimilar and requested guidance on how these issues would influence the comparability exercise, for example, change in expression system, change in formulation from the reference product or if more than 1 manufacturing site for drug product and waiving of phase 3 clinical trials for small biological molecules (e.g. insulin). Product related information for tests on characterization, analytical assessment and product related impurities as well as information on acceptable differences between the RBP and SBP in annexes was also requested.

**Dr Alaa Al-Oballi** (Jordan Food and Drug Administration, Jordan) provided the background on the development of their biosimilars guideline, adopted in 2015 and described the regulatory requirements for biosimilar approval focussing on the comparability exercise which essentially follows standards set by the EMA and WHO. Consequently, only one reference product is allowed throughout the comparability exercise, the biosimilar cannot serve as a reference product by another manufacturer and there is no regulatory requirement to repeat the demonstration of biosimilarity against the reference product once the marketing authorization has been granted. Only indications approved for the risk management plan (RMP) can be granted for the biosimilar. Automatic substitution is not allowed and decision to treat a new patient or switch product requires the opinion of healthcare professionals (HCP) regardless of the product, i.e. biosimilar/reference. Importantly, there is a requirement for a RMP in addition to a pharmacovigilance plan and risk minimisation measures to identify, characterize and minimise a medicine's important risks including Immunogenicity. A fast-track pathway in operation since 2017 which allows products (including biosimilars) authorized in EMA and/or US FDA to be authorized within 60 or 90 days. Products registered as fast track have priority for any post approval variation, if variation approved by the reference health authority. The agency reported that the first draft revision of WHO guidelines was comprehensive, and covered all aspects required to prove similarity (e.g. selection of the reference product, quality non-clinical & clinical evaluation and pharmacovigilance) and provides an overview on waiving of some studies based on proven similarity.

**Ms Watsamon Ondee** (Ministry of Public Health, Thailand) began with a brief background on the development of their national biosimilars guideline in 2013 which is based on EMA guidance. In Thailand, the first biosimilar, Zarzio was approved in 2017. Regarding the guideline, specific issues which require clarification relate to the terminology section which should be expanded to include terms such as stepwise approach, stand-alone product, inferiority trial and totality of evidence, and the scope should be rewritten to address products that are not considered as biosimilars (e.g. vaccines). For clinical trials, it would be useful to include clarification on whether trials such as non-inferiority trial or superiority trial are acceptable or not and for sensitive indication, provide examples or information on how to select the indication in clinical trial. She also urged WHO to provide product specific guidelines.

**Dr Ali Alshahrani** (Saudi Food & Drug Authority, Saudi Arabia) highlighted that the assessment practice for biosimilars in the Saudi FDA is consistent with EMA guidelines. The local Good Pharmacovigilance Practice Guideline is followed, and there is a requirement for a RMP and risk minimisation measures to assess if sufficient - those in place for the reference product are also requested for the biosimilar under evaluation (if the safety concern applies to the biosimilar). He suggested further details to be added in the revised guidelines on different topics, for examples: 1) In a case where there is divergent situation from the normal expected practice, e.g. a biosimilar with a different route of administration from the RBP as seen recently with approval of the subcutaneous route for Remsima in contrast to the intravenous route for the reference product, Remicade; 2) For analytical comparability studies, more information on the age of batches and selection of sample size that is sufficient for similarity evaluation in bioassays taking account of the criticality of quality attribute(s); and 3) In the clinical section, considerations for selecting indication/patient population sensitive for differences in all relevant aspects of safety/efficacy. He also mentioned that the type of product (simple/complex) will be one of the factors informing on the need for a comparative clinical and efficacy trial.

**Dr Patricia Socualaya Sotomayor (**Ministry of Health, Peru) elaborated on the legal and regulatory situation for evaluation of biosimilars in Peru. For biological products including biosimilars (regulaton since 2016), internationally accepted standards like those of the WHO, US FDA and EMA are followed, but there is no position on interchangeability. The terms ‘similar biological product’ and ‘reference biological product’ are used. The reference biological product should be either locally authorized or sourced from a country with strict regulatory standards or from the EU. The approval time-line varies and depends on whether the product has been approved by EMA or another country of high regulatory criteria or has WHO pre-qualification status or is to be assessed locally. Since 2017, an increasing number of biosimilar products have been approved annually (1 in 2017; 9 in 2021 – total 19 products; different product strengths are counted as individual products). A system for pharmacovigilance and suspected adverse reactons is in place with traceability using product trade name, manufacturer, registration number and batch number but there is no official position on interchangeability. There is a need for training of regulators, health care personnel and patients (with focus on Pharmacovigilance). For certain products (pegaspargase, darbepoetin alfa, enzymes and others) where there is no information available on comparability exercises/experience from other countries, difficulties are being encountered for approval as biosimilars.

## 4 Industry perspective on the first draft revision of guidelines

**Dr Martin Schiestl** (Sandoz, representing the International Generic and Biosimilar Medicines Association (IGBA)), gave the biosimilar's manufacturer's perspective. He mentioned that the IGBA welcomed the introduction of the concept of critical quality attribute assessment for biosimilar development, reduction of animal studies, the inclusion of a tailored biosimilar development based on a robust analytical package and a well justified clinical strategy which includes a comparative clinical pharmacokinetic study without a need for a comparative clinical efficacy study. He highlighted topics which could benefit from further clarity. For example, a) for non-local RBPs, considerations on circumstances which justify waiving of bridging data, b) the role of the WHO IS in harmonizing bioactivity and the need for manufacturer's in-house reference standards to ensure consistency during product lifecycle and c) guidance on statistical approaches for supporting evaluation of analytical similarity. Inclusion of a statement reflecting the regulatory position that biosimilarity demonstration is only needed at licensure (and not when post-approval changes occur) and adopting the terminology of ‘biosimilar’ for products approved as per standards in the WHO guidance should also be considered. Finally, lot of public information is available for enhancing knowledge (e.g. agency assessment reports, publications) but implementation workshops have an important role in disseminating knowledge and are essential for strengthening regulatory decision-making and international alignment.

**Dr Virginia Acha** (MSD, representing the International Federation of Pharmaceutical Manufacturers & Associations (IFPMA)) shared the IFPMA position on the waiving of a comparative clinical efficacy trial as conditional upon sound scientific evidence and risk management, based on appropriate criteria and on a case-by-case approach. The guidance should be able to capture the dependence of this strategy on a number of contributing factors e.g., suitability of the target molecule (as noted in the guidelines), the strength of the development plan and the analytical capabilities used by the developer and their ability to provide a convincing case resulting from the “totality of evidence”. IFPMA supports the evolution in the guidance from “step wise” to a “totality of evidence” mindset. In recognition of the important role of *in vitro* studies in biosimilar development, she urged inclusion of detail and clarity on how to assess what *in vitro* studies are fit for purpose (e.g. determination of mechanism of actions, potential for process-related impurities, others) to determine if analytical differences are clinically meaningful, along with the role for validated PD markers in shaping a development program. For immunogenicity, the guidance should address that the risk of immunogenicity depends on context of use (e.g. chronic use. target populations) and that it can be exacerbated following multiple doses and provide guidance on how to assess and respond to severe immune-related reactions. She concluded that WHO must facilitate ongoing international engagement and alignment including through requisite capacity building and technical support measures, ensuring patients globally have access to biosimilars that meet robust and equivalent standards and that IFPMA are willing to support actions for capacity building and international alignment.

**Dr Sandra Yi Cho** (Instituto Butantan, Emerging Biopharmaceutical Manufacturers Network (EBPMN)) provided a brief background of the Emerging Biopharmaceutical Manufacturers Network and one of its members, Instituto Butantan which produces 65% of vaccines and also has a bioindustrial center which has just been completed for manufacturing of monoclonal antibodies for treatment of cancers and autoimmune diseases under contract based on technology transfer from manufacturers with licensed biosimilars. She provided a time-line of SBPs in Brazil since the resolution in 2010 for licensing of biological products. The first SBP, filgrastim, manufactured in 2015 was followed by the approval of etanercept and trastuzumab in 2017, a biosimilar insulin in 2018 and bevacizumab in 2019. She mentioned that the WHO guideline is well organised with distinction of topics, provides clear and detailed information for manufacturers and emphasises the role of the RBP is the comparator for the SBP. In contrast, the national guideline deals mainly with product registration and operational issues e.g. dossier submission and allows registration of SBP products manufactured locally (through tech transfer from a company with approved SBP) with no direct comparison with a RBP.

## 5 Review of the first draft revision

**Dr Hye Na Kang** (WHO secretariat, Switzerland) initiated discussions on the revision of the WHO guideline with respect to the sections on introduction, aim, scope, terminology, scientific considerations and key principles for the licensing of SBPs. The use of ‘direct’ head-to-head (though not necessarily side-by-side) comparisons of a candidate SBPs with a licensed reference product and the deletion of the word ‘step-wise’ were addressed. The definition of ‘reference biological product’ to include its proven quality, efficacy and safety based on its long use was discussed. The use of the term ‘biosimilar’ instead of ‘SBP’ was proposed, since the term ‘SBP’ applies only to biotherapeutic products and the scope of revised guidelines would be expanded. Of note, the WHO survey indicated that the terms ‘biosimilar’, ‘similar biological medicinal product’ and ‘similar biotherapeutic products’ are used interchangeably. For the scope, specific wording on applicability of the guidelines to low molecular weight heparins and recombinant analogues of plasma-derived products but not vaccines and plasma-derived products was considered. The issue of interchangeability was also raised, and it was recognized that the issues associated with the use of biosimilars need to be defined by the individual regulatory authority. She outlined the key principles for licensing of SBPs - High similarity of an SBP to an RBP in structural and functional aspects and nonclinical *in vitro* data is a prerequisite for establishing comparability, with a tailored confirmatory clinical data package required for licensure. A clinical bioequivalence trial with pharmacokinetic (PK) and pharmacodynamic (PD) parameters (if available) in human subjects which includes immunogenicity assessment will typically be a core part of the comparability assessment, unless scientifically justified. The decision for SBP licensure is based on the totality of evidence generated from evaluation of the comparability exercise. If relevant differences between the proposed SBP and the RBP are found at the structural, functional or clinical level, the product is unlikely to qualify as an SBP. As for biosimilarity assessment post-approval, this is only a single event and covered in other documents (e.g. *ref* [[4], [9]]*).*

**Dr Niklas Ekman** (Finish Medicines Agency, Finland) focussed his presentation mainly on the RBP (20 comments) and comparative assessment (n = 30) since these comprised a majority of the comments in the quality section (total 63). He mentioned that a single defined biological product serves as the RBP for the entire biosimilarity exercise and described the RBP's role in biosimilar development, the criteria determining the choice of the RBP and the approach to be taken when the national regulatory authority (NRA) allows use of the same RPB sourced from another jurisdiction (despite availability of local RBP) in clinical studies. In this case, the RPB should be licensed in a jurisdiction with a well-established regulatory framework and bridging studies conducted if needed by the NRA. A comprehensive understanding of the quality and variability of the RBP through detailed characterization of multiple RP batches is necessary to manufacture an SBP product that is highly similar to the RBP along with the usual considerations that apply to manufacturing practice of a biological medicine. A different expression system for SBP is not precluded but the potential of impact on quality (e.g. product-related substances, impurities) has to be considered. Prior to the comparability exercise, a quality attribute criticality assessment and risk assignment (also for development for the manufacturing process) is recommended as it helps in guiding the assessment. For analytical and functional assessment, a thorough characterization of SBP and RBP should be performed using scientifically sound and qualified state-of-the-art techniques and orthogonal methods. The number of RBP and SBP batches to be tested depends on the criticality of the particular quality attribute(s) and the approach for demonstrating similarity. Inclusion of a higher number of batches should increase confidence in results and decrease the risk for a false positive conclusion on similarity. For assessing variability, RBP batches should be procured over an extended time period and the SBP batches tested for comparability should cover commercial scale including process validation batches and those used in the clinical trial(s). Similarity assessment of an SBP is often based on demonstrating that the quality attributes of the SBP batches lie within the predetermined similarity ranges established based on characterization data of RPB batches. For this, various approaches are used e.g. mean ± xSD, min-max range, tolerance intervals. Each statistical approach has specific strengths and weaknesses which should be discussed in the submission and considered in the similarity conclusion. In addition, the manufacturer should justify the relevance of the established similarity ranges and similarity criteria. Any differences detected in quality attributes should be considered as a potential signal for non-similarity and assessed for a possible impact on clinical safety and efficacy.

**Dr Meenu Wadhwa** (National Institute for Biological Standards and Control, UK) provided a brief introduction on international standards (IS), the background to the development of IS for engineered proteins (including monoclonal antibodies) and their unique role in monitoring bioactivity across different products (RBP/SBP) and increasing confidence in product quality [[10], [11], [12]]. She pointed out that for naturally derived proteins such as coagulation factors, hormones (e.g. erythropoietin, follicle-stimulating hormone) where the establishment of the IS (and international units, IU) preceded development of rDNA derived versions, the practice of using the IU for potency assignment, dosage and product labelling is well established and has continued for biosimilars, where applicable. However, the situation is different for non-natural and engineered proteins including monoclonal antibodies (mAbs). In the absence of IS, these products were approved with potency in manufacturer's proprietary units relative to their in-house standard and with product dosing and labelling in mass units. This practice has continued for SBPs and manufacturers are expected to develop well characterized in-house reference standard (2-tiered strategy) for their own product as per regulatory guidance [13]. In all cases e.g. RBP/SBP product, the in-house standard must be calibrated against the IS (where this exists). She explained that WHO International Standards are not medicinal products and serve a different function to the RBP which is essential for biosimilarity. The IS defines the IU of bioactivity for bioassay calibration and has an essential role in the development of suitable assay methods but it cannot be used to determine the product's specific activity or dictate the quality of acceptable SBPs for regulatory purposes (a role assigned only to RBPs). Importantly, the IS allows for an understanding of consistency in bioactivity across batches of a product throughout its life-cycle, provides continuity with respect to the in-house standard and supports transition (change) as the product evolves, facilitates the harmonization of bioactivity across different products (both RBPs and biosimilars) and increases confidence in the quality of available biosimilars [[10], [11], [12]].

**Dr Hans-Karl Heim** (Federal Institute for Drugs and Medical Devices (BfArM), Germany) mentioned that the revision in the non-clinical section aimed to provide a strong focus on *in vitro* (target binding/functional) studies and to introduce flexibility in use of *in vivo* (animal) studies for SBP evaluation. As *in vitro* assays are more specific and sensitive in detecting differences between the RBP and the SBP than *in vivo* animal assays, these should be performed first and the results then used for informing a decision on the need for additional *in vivo* data. The latter should be performed only when specifically needed (represent rare scenario) and in alignment with the 3R (Replace, Reduce, Refine) principles. This change in emphasis has also been included in the SBP mAb guidelines [3]. For *in vitro* testing, a battery of target-binding and cell-based functional assays covering the whole spectrum of pharmaco-toxicological aspects with potential clinical relevance for the RBP should be performed. The studies should be comparative, sufficiently sensitive and cover a concentration range which allows accurate detection of relevant differences in pharmaco-toxicological activity between the SBP and RBP. The testing should include a sufficient number of batches to allow meaningful conclusions on the similarity of SBP and RBP. If evidence from quality and *in vitro* non-clinical data are satisfactory and there is no cause for concern, animal studies are not necessary and can be waived at the discretion of the NRA. However, if a specific need to reduce residual uncertainty prior to clinical trials is identified, additional *in vivo* animal studies may be considered if a suitable animal model exists and the information required cannot be obtained by an alternative approach. If the NRA requires *in vivo* evaluation, studies should be performed in relevant species, e.g., species which are pharmacologically and/or toxicologically responsive to the RBP and in alignment with the 3R principles. For *in vivo* safety studies, a flexible approach which follows 3R principles should be considered.

**Dr Elena Wolff-Holz** (Federal Agency for Vaccines and Biomedicines (PEI), Germany) presented an overview of the sections on clinical aspects, extrapolation, pharmacovigilance, prescribing and labelling. She explained that clinical evaluation is intended to confirm that there are no clinically meaningful differences between the proposed SBP and the RBP. Flexibility has been provided for a stream-lined evaluation where possible with inclusion of a waiver of comparative phase 3 trial if certain conditions are met. A comparative bioequivalence study for PK is always required and should include measurement of PD markers if available as well as safety and immunogenicity. However, the need for a comparative efficacy trial (and the type of trial) is highly dependent on the evidence of biosimilarity and consideration of a multitude of factors (i.e. nature of product and how well it can be characterized, use of orthogonal assays for analytical functional tests, the extent of SBP and RBP similarity, knowledge of the mechanism of actions (MOA) and the extent of *in vitro* evaluation, the clinical history including risk of immunogenicity). For PK, a randomized, two-period, two-sequence, single-dose cross-over PK study in healthy subjects is preferred (using a dose within the therapeutic range and 80–125% equivalence margins) although allowance is provided for a multiple-dose study in patients with sound justification if a single-dose study is not possible. If PK similarity cannot be established (either due to product nature, atypical route of administration, high PK variability), clinical similarity should be supported by PD studies, with immunogenicity, safety and other clinical parameters also examined. Comparative PK/PD studies are often sufficient for clinical similarity if the MOA is well understood and at least one PD marker is linked to efficacy (e.g. an accepted surrogate marker is hemoglobin for epoetin, lactate dehydrogenase for eculizumab). In case an efficacy trial is considered necessary based on the evidence/data generated, an adequately powered, randomized and controlled clinical trial in an homogenous population using an equivalence design (preferred option although both non-inferiority or trials with an asymmetrical margin may be considered if justified) with relevant and sensitive primary end-points is expected. The latter can be the same as used for the RBP originally or different, if well justified. Alternatively, a relevant PD end-point (if a known surrogate of efficacy or linked to the product's MOA) can also serve as the primary end-point. For safety, comparative data from a sufficient number of healthy volunteers and/or patients should be captured. If clinical development is limited to confirmatory PK/PD studies, a risk assessment should be conducted to determine the need to obtain additional safety data for the SBP but it should be noted that impurities necessitate further safety assessments and post-marketing safety monitoring is necessary. Comparative immunogenicity should be investigated in a sensitive population during clinical evaluation with testing using the same assay format and sampling schedule unless the manufacturer can provide a scientific justification that human immunogenicity data are not needed based on a thorough risk assessment (which considers information on the RBP and immunogenicity risk and the factors influencing the SBP's immunogenicity, e.g. product, patient and disease related). Efficacy and safety data from the RBP can be extrapolated to the SBP for all approved indications if the SBP is highly similar to the RBP in terms of analytical and functional properties related to the mechanism(s) of action, supported by clinical data as necessary. The importance of a pharmacovigilance plan for safety monitoring and a legal framework with traceability and identifiable product information for pharmacovigilance as well as for prescribing and labelling was stressed.

## 6 Discussion

The discussions were highly positive, stimulating and generated excellent feedback for further revision. Participants were enthusiastic and appreciated the efforts of the WHO towards producing a scientifically led and well-balanced guideline based on recent developments and alignment where possible with other international and/or national guidelines e.g. US FDA, EMA. Based on the received comments, several major topics which were critical to tailored clinical development emerged for discussion. These have been split into different sections and are listed below:

### 6.1 General points

• ‘Direct’ head-to-head versus side-by-side comparison: It was agreed to keep the term ‘head-to-head’ as real side-by-side analyses (i.e. experiments conducted by the same operator at the same time using the same equipment) are simply not always needed, nor possible to conduct given the number of batches requiring testing. A definition clarifying that the comparison based on historical data is not accepted is included.
• Stepwise approach for biosimilar development: The word ‘stepwise’ was deleted in the draft as this is not followed in practice by developers and this had led to objections. The majority opinion was that ‘tailored’ was more appropriate while acknowledging the evolution from ‘stepwise’ to ‘totality of evidence’ approach. Nevertheless, the concept of stepwise approach is still retained (used in the comparability exercise in ICH Q5E, [14]) since the strategy for clinical development is based on the similarity assessment derived from the analytical and functional data.
• Life-cycle management: following approval, a biosimilar product has its own life-cycle and the concept that a manufacturer is not required to re-establish similarity to the reference product was previously agreed [9,14].
• Preference for terms ‘biosimilars’ and ‘reference products’ instead of SBP and RBP (post meeting note: at the time of the submission of this report for publication, the altered terms were included in the second draft revision of guidelines and subjected for the public consultation).

### 6.2 Scope

There was extensive discussion on this as it was felt that it was best to specify products that were within the scope while also mentioning those that were excluded. Consequently, recombinant analogues of plasma-derived products and low-molecular weight heparins (latter are classed as biologicals in some jurisdictions) were included but vaccines and plasma derived products excluded.

### 6.3 Reference product

This topic generated a lot of comments/discussion and clarification was sought on the following points:

a) whether the strength and pharmaceutical form of the biosimilar should be the same as the RBP in addition to the posology and route of administration since the strength of RBP can alter (due to post-approval changes introduced by the originator) during SBP development. It was explained that in the EU, differences in the strength and pharmaceutical form are allowed, if justified and so the text will be adjusted with inclusion of ‘if justified’ to reflect current practice while also considering its importance from the perspective of dosage and patient safety. The different route of administration (subcutaneous) for the infliximab biosimilar is a post-approval change but again this aspect can be clarified in the guideline.
b) the statement that ‘only one RBP’ should be chosen for a specific SBP for licensing purposes was unclear and further clarity was sought. Does it mean a single product from a single manufacturer? What about different jurisdictions? The basis for selection of a single product from a single manufacturer and its use throughout the comparability exercise along with issues relating to sourcing from another jurisdiction was explained. For a RBP manufactured at different sites, if the license contains different manufacturing sites, it is considered as a single product.
c) use of non-local RBPs and the need for bridging studies. Some participants described the situation in their specific countries e.g., use of non-local RBPs with requirement for bridging studies either due to legal framework or lack of information about product manufacturing sites or approved specifications. Since an obligatory requirement for bridging studies for non-local RBPs was strict but also an impediment for global biosimilar development, the possibility of providing different options including the acceptability by NRA of a non-local RBP without a bridging study and use of public information where feasible/available was urged.

### 6.4 Quality

Regarding the manufacturing process, questions were raised on the number of RBP batches that need to be characterized in order to develop the SBP manufacturing process. It was agreed that specifying a number is not possible – adequate number of RBP batches are needed and data should reflect the variability of the reference product and the assays used. A higher number of batches provides the manufacturer with increased confidence in the data. This question had also surfaced in previous workshops and it was proposed that it should be a topic for an implementation workshop.

On analytical considerations, it had been requested to provide: a) a list of analytical comparability techniques recommended for each type of molecule, b) examples of structural differences, with their respective degrees, that may have a great clinical impact on the product, c) include the validation requirements or refer to the standard guidance for analytical method validation (US FDA and ICH guidance). Regarding points a) and b), the opinion was that provision of this information is difficult in an over-arching guideline. For both, information is available elsewhere in the public domain, either as scientific publications or other documents e.g. public assessment reports, WHO reports on collaborative studies for standards (covering different bioassay techniques) which provide a wealth of useful and valuable information which is difficult to capture in a guideline. For point c), a full validation of methods is not required for characterization methods but state-of -the-art, sensitive and fit for purpose methods are needed.

With regards to comparative analytical assessment, the number of batches of RBP and SBP cannot be specified and it is up to the developer to decide on the number of batches (the same principle as above applies regarding specifying the number). It should be noted that appropriate choice and use of statistical methods is essential for establishing similarity ranges. Different statistical intervals can be used to establish similarity ranges e.g. mean ± x SD, the min-max range and tolerance intervals. For the x-sigma approach, the use of 2 as a multiplier (the 2SD rule) was proposed and extensively discussed. There was agreement that the approach adopted for similarity should be justified and the criticality of the quality attributes should be considered while establishing ranges but it would be best to abstain from specifying a number (2SD may be too restrictive) – if a quality attribute is of high criticality, the multiplier should typically be lower than that of a less critical quality attribute. It was pointed out that the min-max approach has a tendency of high risk for a false negative result (i.e. associated with a high risk of concluding non-similarity despite the underlying data supporting a similarity claim) whereas similarity ranges based on tolerance intervals require a high number of RBP batches and appropriate parametrization. Inclusion of further detail in the guidelines on the approaches and their caveats was proposed.

A difficult and controversial topic was the issue of international standards. While the use of International Standards for assay development, for independent SBP/RBP potency monitoring SBP/RBP or additional external control samples was supported, concern was expressed regarding the requirement to use international standards as primary standards for SBP manufacturers since the potency of the SBP should be aligned with the RBP and use of the IS would result in a systematic difference between the potency of the RBP and the SBP. It was explained that the role of the IS in potency assignment varies depending on the product (presentation above) and in all situations (even where new IS are developed), adherence to biosimilarity principles is expected (potency of the biosimilar should be aligned to the RBP). Another contentious issue which emerged was the terminology which refers to the IS as the ‘primary standard’ as per WHO IS principles while for the manufacturer, the in-house reference standard (2-tiered strategy) serves as the primary standard (as per ICHQ6B, [13]). It was agreed that this wording would be reconsidered for the text but importantly, it was clarified that the principles of WHO biological standardization would prevail and the expectation is that all manufacturer's standards (RBP/SBP) are calibrated against the IS when available. It was agreed that the text would be rewritten in sections on international standards and biological activity to reflect the discussions and satisfy all concerns relating to the IS, potency assessment and the issue with the ‘primary standard’. Additionally, the distinction in the role and use of the IS (and its limitations) and the reference product would be further elaborated in the revised draft.

### 6.5 Non-clinical

This section was fairly straightforward and did not stimulate any discussion. A limited number of comments aimed towards consistency in terminology and improvement in wording rather than a principal change in content were received and these were accepted in a majority of instances. Regarding the question on whether WHO can recommend NRAs to revise the local regulations to allow the possibility of waiving *in vivo* studies based on data from *in vitro* studies, it was explained that WHO standards are recommendations (and advise) and cannot be imposed/enforced and the implementation of any guidance whether full/partial is at the NRA's discretion.

### 6.6 Clinical

The term ‘phase 3’ in the sentence ‘A comparative clinical *phase 3* trial *will not* be necessary if sufficient evidence of biosimilarity can be drawn from other parts of the comparability exercise’ was considered inappropriate by most with preference for the use of adequately powered efficacy trial as this is the terminology often used in regulatory guidance on biosimilars. Additionally, different views on ‘will not’ versus ‘may not’ were presented but since ‘may not’ leads to ambiguity and not a definitive way forward which is really needed for the context in which it is being applied, the conclusion was to adopt ‘will not’ and strengthen the specific criteria which would enable decision-making.

One of the major discussion points was the specific criteria which need to be considered while assessing the need and type of comparative clinical phase 3 trials. The need for functional data as part of the analytical characterization was clearly important given the criticality of these specific criteria (for both the developer and NRA). However, it was stressed that more emphasis on generating functional data relevant to mechanism of action (and in some cases, for plausible MOA) as part of the analytical characterization using ‘suitable and sensitive orthogonal assays’ was clearly essential (although the contribution of each mechanism of action to the observed clinical effect is not relevant as long as it can be measured) for tailored clinical development. The inclusion of ‘clinical history of RBP (including immunogenicity)’ as one of the criteria was questioned by some but it was clarified that this aspect relates to concern on the potential risk of immunogenicity (based on information on the RBP and presence of any novel impurities and/or excipients in SBP) and is highly relevant. The general opinion was that this section should be sufficiently descriptive with requirements clearly stated with due consideration given to the need for scientific and adequately documented justification based on sufficient evidence of biosimilarity from other parts of the comparability exercise.

For comparative PK/PD studies, an explanation regarding the selection and choice of PD markers to be used was sought and specific criteria will be included to reflect that the PD marker should be representative of the MOA of the drug, sensitive to differences and measurable using a validated assay while encouraging use of multiple PD markers and allowing assessment of sensitive PD end-points (even if relevant PD measures are not available) to reduce residual uncertainty [15].

A point of major concern based on received comments and voiced by participants was whether the safety data from the limited clinical assessment (whether PK/PD studies or stand-alone PK study in a scenario where no PD markers exist) will be adequate given that the significance of safety including immunogenicity should not be underestimated. It was explained that the intention, as stated in the guidelines, is that safety data are captured from a sufficient number of subjects throughout clinical development from PK/PD studies (or stand-alone PK) and also in clinical efficacy trials where conducted. If stand-alone comparative PK studies are performed (if no PD marker exists), these studies can be extended over a long duration or separate safety/immunogenicity studies undertaken based on the need for additional safety/immunogenicity data on the biosimilar as stated in the safety section of the guidelines. The extent of data required to characterize the safety profile of the SBP is determined by the applicant based on knowledge of the adverse drug reactions compared with RBP, evidence of similarity of the SBP and the RBP and a thorough risk assessment (e.g., presence of any novel impurities and/or excipients in SBP). High similarity in analytical characterization and PK/PD profiles of the biosimilar and RP and similar/low risk of biosimilar compared with RBP could provide sufficient reassurance obviating the need for further safety data. Examples of such products are insulin, teriparatide, filgrastim or somatropin but complex products such as mAbs and mAb-like products (fusion proteins) will increasingly fall into this category. However, if the SBP contains novel impurities, additional safety data or scientific justification for lack of data are needed, but in such instances, feedback should be sought from regulators.

A related question ‘Are comparative immunogenicity data collected in the PK similarity study sufficient/adequate to demonstrate similarity of immunogenicity between the proposed SBP and the RBP?’ resulted in varied opinion on the adequacy of limited immunogenicity studies with some contentious (mainly conservative) views expressed. Nevertheless, it was agreed that for immunogenicity testing, a similar rationale (as for safety testing) applies with additional consideration given to the type of product while evaluating the risk. It is acknowledged that the risk is higher for a product that has an endogenous non-redundant counterpart (e.g., epoetin) with requirement for clinical studies and real time testing for neutralizing ADAs whereas for biological substances such as insulin, somatropin, filgrastim, teriparatide, where substantial evidence suggests that immunogenicity has no impact on product safety and efficacy, immunogenicity studies may not be necessary (if high biosimilarity and similar/low risk assessment of SBP vs RBP). This may also apply to other products, including mAbs but regulatory opinion is urged. Inclusion of a requirement for scientific justification for waiving a safety/immunogenicity study was suggested. Despite disparate opinions on the need for immunogenicity studies including EMA guidance which requires clinical immunogenicity studies for biosimilar insulins and somatropin, evidence to date for the examples cited is sound and unconflicted refuting the need for any immunogenicity studies. This is supported by regulatory experience from recent examples of biosimilar approvals where the immunogenicity profile observed in the PK trial is similar to the data from confirmatory pivotal trials.

In cases where immunogenicity testing reveals a questionable difference in immunogenicity (lower/higher), an analysis of the underlying cause, and data and justification to support a claim that the difference is not clinically relevant is expected.

For biosimilar approval, totality of evidence is needed and there should be no residual uncertainty.

### 6.7 Pharmacovigilance

For pharmacovigilance and adverse reaction reports, proper information on traceability is important. Deletion of information relating to ‘country of origin’ was initially proposed but taking into account the usefulness of this information, it was agreed that the report should include International Nonproprietary Names (INN) as well as proprietary (brand) name, manufacturer's name, and lot number and country of origin.

## 7 Conclusion and way forward

a. It was clear that the revised draft guideline had been well received based on the excellent and positive feedback from various stakeholders, e.g. regulators, industry etc.
b. Comments received indicated that certain parts in various sections in the guideline were unclear and required modifications. In addition to the topics highlighted above, these were mainly related to the terminology used (e.g. head-to head vs side-by-side, SBP instead of the commonly used ‘biosimilar’, stepwise vs tailored, phase 3 trial vs efficacy trial) and the scope.
c. Expansion of certain sections (e.g. reference biological product, statistical approaches for similarity in the analytical assessment, WHO international standard, clinical trial designs, in particular, non-inferiority trials and those with an asymmetrical margin) and clarity on scenarios in which immunogenicity studies may not be necessary was urged.
d. There was general agreement on the proposed text and modifications among the participants and a concrete way forward emerged for flexibility and a further revision of the draft guideline in relevant areas. For example, for the RBP, multiple bridging studies in various regions with different licensed comparators depending on where approval is being sought, is a major challenge. In some cases, it is a legal requirement so difficult to harmonize but various options will be included to allow for decision-making by the NRA.
e. As part of the consultation, representatives from participating NRAs reported that their national requirements have been defined based on principles described in the WHO guidelines and those of leading regulatory agencies. However, some countries lack national guidance while others are in the process of developing guidance as highlighted by the WHO survey. For these countries/regions, the stream-lined revised guidelines and associated implementation workshops will provide the impetus and help towards establishing guidance and regulatory capacity-building and strengthening where needed. There was consensus from all stakeholders that information sharing needs to be enhanced at the global level and urgent steps taken to implement the principles in the WHO guidelines. This would improve consistency and regulatory decision making at the national level and provide rapid access to safe and effective medicines. Importantly, successful implementation would promote regulatory convergence and harmonization.
f. Several topics were identified for information sharing either via implementation activities and/or through training workshops, case-studies, development of learning tools, publications etc. These are listed below:
  • Tailored approach
  • Number of RBP batches for the development of SBP manufacturing process
  • Analytical considerations, e.g. list of (advanced) techniques, examples of structural differences affecting clinical performance, validation requirement (Consider using the materials from the International Pharmaceutical Regulators Programme, Biosimilars Working Group)
  • The role of WHO international standards and their use
  • Flexible approach in nonclinical study, e.g. stepwise (*in vitro* affects to waiving *in vivo* study)
  • Provide more specific information about nonclinical *in vitro* assays fit-for-purpose for evaluation of SBPs
  • Factors or criteria which influence the need (and type) of comparative safety and efficacy trial
  • Risk-assessment for safety and immunogenicity and the data package required for justification.
  • Differences in immunogenicity (low or high): how to justify?

Overall, the general opinion was that the revised guideline is well-drafted, has taken into account the scientific advances and experience gained since adoption of the previous version while also anticipating future opportunities. NRAs are monitoring further progress of the guidelines. While some are keen to adapt their regulatory practice/guidelines to be consistent with WHO guidance, others are concerned and cautious of the heavy reliance on the quality and non-clinical data (and monitoring how the situation unfolds in the EU) and the flexible and tailored clinical approach for biosimilar development outlined in the guidelines. Undoubtedly, this offers an opportunity for biosimilar manufacturers while recognising that the totality of evidence concept applies for product approvals. Extensive training via WHO workshops to allow consistent and effective implementation to avoid misunderstandings and foster knowledge is necessary for strengthening regulatory decision-making and international alignment.

## Disclaimer

The authors alone are responsible for the views expressed in this article and they do not necessarily represent the views, decisions or policies of the institutions with which they are affiliated.

## Funding

The Ministry of Health and Welfare, Republic of Korea provided the fund to WHO for the open access of this article through a voluntary contribution.

## Declaration of competing interest

The authors have completed the WHO declaration of interests form. It is required that experts serving in an advisory role disclose any circumstances that could give rise to a potential conflict of interest related to the subject of the activity in which they will be involved.

## References

[1] (2013). WHO expert committee on biological standardization: sixtieth report.

[2] Resolution WHA67.21 (2014). http://apps.who.int/gb/ebwha/pdf_files/WHA67/A67_R21-en.pdf.

[3] (2017). WHO expert committee on biological standardization: sixty-seventh report.

[4] (2018). WHO Questions and Answers: similar biotherapeutic products. Complementary document to the WHO Guidelines on evaluation of similar biotherapeutic products (SBPs).

[5] (2021). WHO expert committee on biological standardization: report of the seventy-second and seventy-third meetings.

[6] Kang H.-N., Thorpe R., Knezevic I. (2020). The regulatory landscape of biosimilars: WHO efforts and progress made from 2009 to 2019. Biologicals.

[7] Kang H.-K., Thorpe R., Knezevic I. (2021). Regulatory challenges with biosimilars: an update from 20 countries. Ann N Y Acad Sci.

[8] Kang H.-N., Thorpe R., Knezevic I. (2021). Biosimilars – status in July 2020 in 16 countries. GaBi Journal.

[9] (2018). WHO expert committee on biological standardization: sixty-eighth report.

[10] Thorpe R., Wadhwa M. (2011 Sep). Intended use of reference products & WHO International Standards/Reference Reagents in the development of similar biological products (biosimilars). Biologicals.

[11] (2016). WHO expert committee on biological standardization: sixty-sixth report.

[12] Prior S., Metcalfe C., Hufton S.E., Wadhwa M., Schneider C.K., Burns C. (2021 Mar). Maintaining 'standards' for biosimilar monoclonal antibodies. Nat Biotechnol.

[13] (1999). Specifications: test procedures and acceptance criteria for biotechnological/biological products. ICH harmonised tripartite guideline Q6B. Geneva: international conference on harmonisation of technical requirements for registration of pharmaceuticals for human use. https://database.ich.org/sites/default/files/Q6B%20Guideline.pdf.

[14] (2004). Comparability of biotechnological/biological products subject to changes in their manufacturing process. ICH harmonised tripartite guideline Q5E (step 4). Geneva: international conference on harmonisation of technical requirements for registration of pharmaceuticals for human use. https://database.ich.org/sites/default/files/Q5E%20Guideline.pdf.

[15] Li J., Floreian J., Campbell E. (2020 Jan). Advancing biosimilar development using pharmacodynamic biomarkers in clinical pharmacology studies. Clin Pharmacol Ther.

